# Dietary Ferrous Sulfate Enhances Resistance to *Vibrio splendidus*-Induced Skin Ulceration in *Apostichopus japonicus* via Immune and Antioxidant Modulation

**DOI:** 10.3390/pathogens14090952

**Published:** 2025-09-22

**Authors:** Ye Tian, Kaihao Zhao, Xiaonan Li, Lina Cao, Lingshu Han, Chong Zhao, Jun Ding

**Affiliations:** 1 Key Laboratory of Northern Aquatic Germplasm Resources and Genetic Breeding in Liaoning Province, Dalian Ocean University, Dalian 116023, China; 2 Key Laboratory of Mariculture & Stock Enhancement in North China’s Sea, Ministry of Agriculture and Rural Affairs, Dalian Ocean University, Dalian 116023, China; 3 Dalian Jinshiwan Laboratory, Dalian 116034, China

**Keywords:** *Apostichopus japonicus*, ferrous sulfate, *Vibrio splendidus*, immunity, antioxidant response, skin ulceration, feed additive, disease resistance, sea cucumber aquaculture

## Abstract

The sea cucumber (*Apostichopus japonicus*) is a commercially important marine species. However, its survival is increasingly threatened by frequent outbreaks of Skin Ulceration Syndrome caused by *Vibrio splendidus*. This study evaluated the effects of dietary supplementation with ferrous sulfate (FeSO_4_) at two concentrations (0.5% and 1%) over short-term (21 days) and long-term (56 days) feeding periods on immune defense, antioxidant capacity, and resistance to *V. splendidus* infection. Key parameters measured included survival rate, cellular immune activity, antioxidant enzyme levels, and expression of immune-related genes. Long-term (56 days) supplementation with 1% FeSO_4_ significantly improved survival after infection (90 ± 4.7%). Phagocytic activity and respiratory burst were enhanced by approximately ~1.9-fold and ~1.8-fold, respectively (*p* < 0.05). The expression of *sod*, *ferritin*, and *hsp70* genes was upregulated by ~2.1-fold, ~2.0-fold, and ~1.6-fold, respectively (*p* < 0.05). These results indicate strengthened cellular immunity and antioxidant capacity. Long-term (56 days) supplementation with 0.5% FeSO_4_ increased lysozyme activity (~1.3-fold) and *c3* expression (~4-fold) (*p* < 0.05), thereby enhancing humoral immunity. In contrast, short-term (21 days) supplementation increased ACP and AKP activities by approximately ~2-fold each, and LZM activity by ~1.2-fold (*p* < 0.05). However, it did not significantly improve survival, indicating limited protective effects. Overall, 56-day dietary supplementation with FeSO_4_, particularly at 1%, effectively enhances immune and antioxidant responses in *A. japonicus.* This supplementation represents a promising strategy for preventing *V. splendidus*-induced skin ulceration in aquaculture.

## 1. Introduction

The sea cucumber *Apostichopus japonicus* is a commercially valuable marine species. It is highly regarded in both the food and pharmaceutical industries due to its rich content of proteins, polysaccharides, trace elements, and other bioactive compounds [1,2]. In recent years, sea cucumber aquaculture has been severely affected by frequent outbreaks of Skin Ulceration Syndrome (SUS), which cause high mortality and threaten the sustainable development of the sector [3,4]. SUS is characterized by skin ulcerations, internal organ damage, and, in severe cases, death. The disease progresses rapidly, spreads quickly, and has a high mortality rate, causing considerable economic losses in aquaculture [5]. Research has identified *V. splendidus* as the primary pathogen responsible for SUS. This bacterium exhibits strong infectivity and pathogenicity and is difficult to control, posing a serious threat to the health of sea cucumber farms [6,7].

Current control measures for SUS in sea cucumbers primarily involve pharmaceutical treatments, immunostimulant supplementation, and environmental regulation. However, these approaches have notable limitations. For instance, the frequent use of antibiotics can lead to pathogen resistance and potential drug residue issues, jeopardizing food safety and the environment [8,9]. Although immunostimulants can temporarily enhance the immune defenses of sea cucumbers, their effects are often unstable and short-lived [10,11,12]. Environmental regulation strategies are resource-intensive and require specialized facilities. They also involve complex operational procedures, which makes their widespread implementation difficult [13]. Thus, there is an urgent need for a safe, effective, and environmentally friendly control strategy.

Recent studies suggest that iron supplementation, such as with ferrous sulfate (FeSO_4_), enhances immune function in teleosts like grass carp and inhibits *Vibrio harveyi*, highlighting its preventive potential [14,15]. In sea cucumbers, iron plays a central role in modulating innate immunity through its involvement in redox processes. Iron availability regulates ROS production in coelomocytes, influencing antibacterial responses and controls ferroptosis, a form of cell death that restricts pathogen growth [16,17]. Ferritin stores labile iron and contributes to the acute-phase response, while iron mobilization via ferritinophagy helps balance iron levels during infection [18,19]. Additionally, iron-linked redox regulators, including miRNA pathways, fine-tune immune responses by modulating ROS and effector activity [20]. Building on this, we hypothesize that long-term supplementation with FeSO_4_ will enhance the immune function and antioxidant capacity of sea cucumbers, influencing the expression of antioxidant genes (e.g., *sod* and *ferritin*), as well as phagocytic cell activity, respiratory burst, and related enzyme activities. Therefore, this study investigates the effects of different feeding regimes and dosages of ferrous sulfate (FeSO_4_) on immune responses, antioxidant enzyme activities, and the expression of immune-related genes in *A. japonicus*. The aim is to evaluate its potential as a sustainable disease management strategy. Additionally, the study seeks to deepen the theoretical understanding of SUS and offer practical approaches for disease management in *A. japonicus*. These insights aim to support the long-term sustainability of sea cucumber aquaculture.

## 2. Materials and Methods

### 2.1. Experimental Materials

#### 2.1.1. Sea Cucumbers

The experiment was conducted at the Key Laboratory of Mariculture & Stock Enhancement in North China’s Sea, Ministry of Agriculture and Rural Affairs (Dalian, China). Healthy sea cucumbers were obtained from a farm in Dalian. After a one-month acclimatization period, 190 healthy sea cucumbers with an average weight of (20 ± 3.7) g and uniform size were selected for the study. Before the experiment, the sea cucumbers were placed in 40 L tanks, with 10 individuals per group, and acclimatized for an additional week. Once their health status was confirmed to be stable, they were used for the formal experiment.

#### 2.1.2. Pathogenic Strain

The pathogenic strain used in this study was kindly provided by Dalian Ocean University. A single *V. splendidus* strain was selected and purified. The strain was inoculated into 2216E liquid culture medium and incubated in a shaking incubator at 28 °C and 200 rpm for 12–16 h. It was allowed to grow until it reached the logarithmic phase (OD600 = 0.5–0.7, corresponding to a bacterial concentration of approximately 1 × 10^7^ CFU/mL). This bacterial suspension was then used for the infection trials [21].

#### 2.1.3. Feed Preparation

The basic feed mixture for all groups was prepared by combining marine mud with compounded feed at a mass ratio of 2:1. This mixture served as the basal diet for the control group. In the experimental groups, ferrous sulfate (FeSO_4_) was added to the basal diet at concentrations of 0.5% and 1.0%, respectively. The mixtures were thoroughly blended to ensure even distribution.

#### 2.1.4. Reagents

For the determination of coelomocyte phagocytic and respiratory burst activities in sea cucumbers, the following reagents were used: EGTA, NaCl, KCl, Tris-HCl, neutral red solution, glacial acetic acid, absolute ethanol, PBS buffer, NBT reaction solution, methanol, KOH, and DMSO. All reagents were purchased from Beyotime Biotechnology (Shanghai, China). The activities of lysozyme (LZM), superoxide dismutase (SOD), acid phosphatase (ACP), and alkaline phosphatase (AKP) were determined using commercial assay kits (Nanjing Jiancheng Bioengineering Institute, Nanjing, China). RNA extraction and quantitative real-time PCR were performed using TRIzol reagent, chloroform, isopropanol, 75% ethanol, RNase-free water, FastKing One-Step Genomic cDNA Synthesis PreMix Kit, and FastReal SYBR Green Quantitative PCR PreMix Kit. All this purchased from Tiangen Biotech (Beijing, China).

### 2.2. Experimental Design

Short-Term Feeding Trial: Ninety sea cucumbers were randomly assigned to three dietary treatments: a control group (basal diet), a 0.5% FeSO_4_ group, and a 1% FeSO_4_ group, with FeSO_4_ supplemented in the diets. Each group included three replicates, with 10 individuals per replicate. After three weeks of feeding, the animals were exposed to a pathogen challenge, along with those in the long-term feeding trial. During the trial, the water temperature was maintained at 16–18 °C, the pH at 8.00 ± 0.15, and salinity at 30‰. Continuous aeration (24 h per day) was provided, and feeding occurred daily at 18:00 with a daily ration of 5% of the sea cucumbers’ body weight. Residual feed and feces were removed at 9:00 the following day and 50% of the seawater was replaced.

Long-Term Feeding Trial: Another 90 sea cucumbers were equally divided into three groups with the same dietary treatments: control (basal diet), 0.5% FeSO_4_, and 1% FeSO_4_. Each group contained three replicates, with 10 individuals per replicate. After eight weeks of feeding with FeSO_4_-supplemented diets, the animals were challenged with *V. splendidus*. Feeding methods and water quality maintenance were consistent with those used in the short-term trial.

Infection Challenge: A pre-experiment was conducted to determine the 14-day median lethal dose (LD50) of *V. splendidus.* Ninety sea cucumbers were randomly assigned to three groups (30 individuals per group) and exposed to three concentrations of *V. splendidus* suspension (1 × 10^3^, 1 × 10^5^, and 1 × 10^7^ CFU/mL) for 14 days. Daily mortality was recorded, and LD_50_ was estimated using probit analysis, following established protocols. The calculated 14-day LD_50_ was 1 × 10^5^ CFU/mL. Following the feeding trials, 40 mL of a *V. splendidus* suspension (1 × 10^7^ CFU/mL) was added to each 40 L tank, raising the seawater concentration to 1 × 10^5^ CFU/mL. The sea cucumbers were continuously exposed for 14 days, with half of the water replaced daily. After each water change, fresh *V. splendidus* suspension was reintroduced to maintain the target concentration. Feed was provided daily throughout the infection period, and the health status of the sea cucumbers was monitored daily.

### 2.3. Sample Collection

After the infection challenge, 3 sea cucumbers (n = 3) were randomly selected from each experimental group. Coelomic fluid was extracted from the sea cucumbers using a sterile syringe and divided into two portions. One portion was used to measure enzyme activities (LZM, SOD, ACP, and AKP) after centrifugation. The other portion, mixed with an equal volume of anticoagulant (0.02 mol/L EGTA, 0.48 mol/L NaCl, 0.019 mol/L KCl, 0.068 mol/L Tris-HCl, pH = 7.6), was used to assess phagocytic cell activity and respiratory burst.

Following coelomic fluid extraction, the sea cucumbers were dissected to obtain the internal body wall. The tissue was rapidly frozen in liquid nitrogen and stored at −80 °C for later RNA extraction, which was subsequently used for real-time quantitative PCR analysis.

### 2.4. Measurement of Phagocytic and Respiratory Burst Activity

Phagocytic Cell Activity Assay [22]: Anticoagulated coelomic fluid was added to each well of a 96-well plate, with 100 µL per well and 3 technical replicates for each sample. The cells were incubated at 18 °C for 30 min to allow adhesion. After incubation, the supernatant was removed without disturbing the adherent cells. Then, 100 µL of 0.001 mol/L neutral red solution was added to each well, and the plate was incubated for 30 min. The solution was aspirated, and the wells were washed three times with PBS. Next, 100 µL of cell lysis buffer (a 1:1 mixture of glacial acetic acid and absolute ethanol) was added, and the plate was incubated for 20 min to release the neutral red dye. Finally, absorbance was measured at 540 nm using a microplate reader.

Respiratory Burst Activity Assay [23]: Anticoagulated coelomic fluid was added to each well of a 96-well plate, with 100 µL per well and 3 technical replicates for each sample. The cells were incubated at 18 °C for 1 h to allow adhesion. After incubation, the supernatant was removed, and 200 µL of 0.2% NBT reaction solution was added to each well. The plate was incubated for 30 min, and the solution was then aspirated. To terminate the reaction, 100 µL of methanol was added and incubated for 10 min, followed by three washes. Next, 120 µL of 2 mol/L KOH and 140 µL of DMSO were added to each well, and the plate was mixed. Absorbance was measured at 630 nm.

### 2.5. Immune and Antioxidant Enzyme Activity Assays

The activities of acid phosphatase (ACP), alkaline phosphatase (AKP), lysozyme (LZM), and superoxide dismutase (SOD) were measured using the coelomic-fluid supernatant obtained from the collected samples (n = 3), with three technical replicates per sample. LZM activity was determined using a turbidimetric method, SOD activity by the xanthine oxidase method, ACP activity by the phenyl phosphate method, and AKP activity by the phenyl phosphate method. All assays were performed strictly according to the instructions provided with the commercial kits from Nanjing Jiancheng Bioengineering Institute (Nanjing, China).

### 2.6. RNA Extraction and Real-Time Fluorescence Quantitative PCR (qPCR)

#### 2.6.1. RNA Extraction

Three sea cucumbers (n = 3) were randomly selected from each group, and 100 mg of internal body wall tissue was collected. Total RNA was extracted using the TRIzol method. The tissue was homogenized in 1 mL of TRIzol, incubated for 5 min, and mixed with 0.2 mL of chloroform. After shaking and a 5 min incubation, the sample was centrifuged at 12,000 rpm for 10 min. The upper aqueous phase was transferred to a new tube, and an equal volume of isopropanol was added to precipitate the RNA. After 10 min, the sample was centrifuged again, and the supernatant was discarded. The RNA pellet was washed with 75% ethanol and centrifuged for 5 min. After air-drying, the RNA was dissolved in RNase-free water [24]. RNA integrity was assessed by agarose gel electrophoresis, and purity and concentration were measured using a NanoDrop 2000 spectrophotometer (Thermo, USA) [25]. RNA samples were stored at −80 °C.

#### 2.6.2. cDNA Synthesis

cDNA was synthesized from the RNA using the FastKing One-Step Genomic cDNA Synthesis PreMix Kit from TianGen Biotech (Beijing) Co., Ltd. (Beijing, China).

#### 2.6.3. qPCR Reaction

Quantitative real-time PCR (qRT-PCR) was performed using FastReal SYBR Green Master Mix (Tiangen Biotech, Beijing, China). The *β-actin* gene was used as the internal reference. Six target genes were selected: *sod*, *ferritin*, *c3*, *tlr3*, *hsp70*, and *lyz*. All primers were synthesized by Sangon Biotech (Shanghai, China) and are listed in Table 1.

Four primer pairs (*tlr3*, *hsp70*, *lyz*, and *β-actin*) were adopted from previously published studies (citations provided in Table 1), where they had been validated in *A. japonicus.* The remaining primer pairs (*ferritin*, *sod*, and *c3*) were newly designed in this study using Primer Premier 5.0, based on sequences available in GenBank. Their specificity was confirmed by BLAST analysis, melt curve analysis, and agarose gel electrophoresis, all of which showed single amplicons of the expected size.

To statistically evaluate the interaction effects between FeSO_4_ concentration and feeding duration (short-term vs. long-term) on gene expression in sea cucumbers, all relative expression levels were normalized using a unified reference baseline. Specifically, the control group from the short-term feeding trial was designated as the global reference. ΔCt values were first calculated for all samples, and relative expression was then determined using the 2^–ΔΔCt^ method. The average ΔCt of the short-term control group served as the calibrator for ΔΔCt calculations. This normalization strategy ensured comparability of relative expression levels across all treatment groups and satisfied the requirements for subsequent two-way ANOVA analysis [30].

### 2.7. Statistical Analysis

All statistical analyses were performed using GraphPad Prism version 10.0. Prior to ANOVA, data normality and variance homogeneity were checked in GraphPad Prism using the Shapiro–Wilk test and Levene’s test, respectively, and both assumptions were satisfied. To assess the effects of FeSO_4_ concentration, feeding duration (short-term vs. long-term), and their interaction on various physiological, biochemical, and molecular parameters of sea cucumbers, two-way analysis of variance (Two-way ANOVA) was employed to detect differences among treatment groups. For factors showing significant main effects or interactions (*p* < 0.05), post hoc comparisons were conducted using Tukey’s Honestly Significant Difference (Tukey’s HSD) test. A significance level of *α* = 0.05 was set for all hypothesis testing. Differences were considered statistically significant at *p* < 0.05 and non-significant at *p* > 0.05.

## 3. Results

### 3.1. Survival Rate

As shown in Table 2, the survival rates of sea cucumbers in the short-term and long-term control groups were 50 ± 10% and 53 ± 12.5%, respectively, with no significant difference between them (*p* > 0.05). The relatively high standard deviations observed in the control groups suggest greater individual variation in stress tolerance and baseline survival under the experimental conditions. In the FeSO_4_-treated groups, survival rates exhibited an increasing trend with both higher FeSO_4_ concentrations and longer feeding durations. Notably, the long-term 1% FeSO_4_ group showed a significantly higher survival rate (90 ± 4.7%) compared to all control and other treatment groups (*p* < 0.05). No significant differences in survival rates were observed among the other FeSO_4_-treated groups (*p* > 0.05).

### 3.2. Phagocytic Cell Activity in Coelomic Fluid

As shown in Table 2, no significant difference in phagocytic activity was observed between the short-term and long-term control groups (*p* > 0.05). In the short-term FeSO_4_-treated groups (0.5% and 1%), phagocytic activity was not significantly higher than that of the control group (*p* > 0.05). In contrast, the long-term 1% FeSO_4_ group exhibited the highest phagocytic activity, which was significantly higher than all short-term groups (*p* < 0.05) and also higher than the long-term 0.5% FeSO_4_ group, although the difference between the two long-term groups was not statistically significant (*p* > 0.05).

### 3.3. Respiratory Burst Activity in Coelomic Fluid

As shown in Table 2, the respiratory burst activity in the long-term 1% FeSO_4_ group was significantly higher than that in all other groups (*p* < 0.05). No significant difference was observed between the long-term 0.5% group and the control group (*p* > 0.05); however, the long-term 0.5% group exhibited significantly higher respiratory burst activity than the short-term treatment groups (*p* < 0.05). Both the short-term 1% and 0.5% groups showed relatively low respiratory burst activity, which was significantly lower than that of the long-term high-dose group (*p* < 0.05).

### 3.4. Immune and Antioxidant-Related Enzyme Activities

As shown in Table 3, FeSO_4_ treatment had a significant effect on AKP activity (*p* < 0.05). In the short-term feeding groups, both 0.5% and 1% FeSO_4_ significantly increased AKP activity compared to their respective control groups, with the short-term 0.5% group showing the highest activity among all groups (*p* < 0.05). In contrast, long-term feeding with 0.5% and 1% FeSO_4_ significantly reduced AKP activity, which was notably lower than that of both control groups and short-term treatment groups (*p* < 0.05).

ACP activity also differed significantly among the treatment groups (*p* < 0.05). Short-term FeSO_4_ feeding significantly elevated ACP activity, with the 0.5% group displaying the highest value. Although no significant difference was found between the 0.5% and 1% short-term groups, both were significantly higher than their respective controls (*p* < 0.05). Conversely, long-term FeSO_4_ treatment markedly suppressed ACP activity. No significant difference was observed between the 0.5% and 1% long-term groups, but both were significantly lower than all short-term groups and the long-term control (*p* < 0.05).

SOD activity showed significant differences among some treatment groups (*p* < 0.05). Both the short-term 1% FeSO_4_ group and the long-term 1% group had significantly higher SOD activity compared to their respective controls (*p* < 0.05). The long-term 1% group exhibited the highest SOD activity across all treatments (*p* < 0.05). No significant differences in SOD activity were observed among the remaining groups (*p* > 0.05).

FeSO_4_ treatment also had a significant impact on LZM activity (*p* < 0.05). All FeSO_4_-fed groups exhibited significantly higher LZM activity than the control groups (*p* < 0.05). Among them, the long-term 0.5% group showed the highest activity, which was significantly greater than that of all other treatment groups (*p* < 0.05), indicating a strong immunostimulatory effect. No significant difference was observed between the short-term 0.5% and 1% groups (*p* > 0.05), although both were significantly higher than the short-term and long-term control groups (*p* < 0.05). The long-term 1% group showed slightly elevated LZM activity compared to the control, but the difference was not statistically significant when compared with other treatment groups (*p* > 0.05).

### 3.5. Gene Expression Analysis

**Figure 1:**
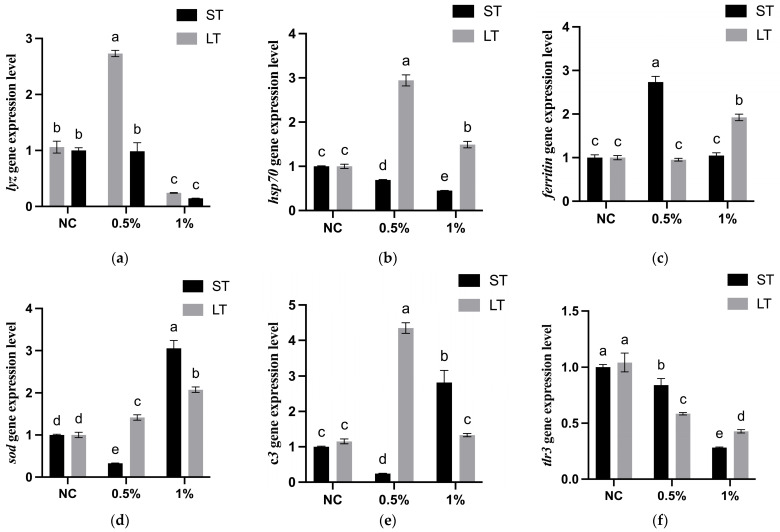
Relative expression levels of immune-related genes in *A. japonicus* after FeSO_4_ supplementation. ST = short-term; LT = long-term. Gene abbreviations: (**a**) *lyz* = lysozyme, (**b**) *hsp70* = heat shock protein 70, (**c**) *ferritin* = ferritin, (**d**) *sod* = superoxide dismutase, (**e**) *c3* = complement component 3, (**f**) *tlr3* = Toll-like receptor 3. Data are presented as mean ± SD (n = 3). Different letters above the bars indicate significant differences among treatments (*p* < 0.05).

As shown in Figure 1a, *lyz* expression was significantly downregulated in both short-term and long-term 1% FeSO_4_ groups (*p* < 0.05). In contrast, long-term feeding with 0.5% FeSO_4_ markedly upregulated *lyz* expression (*p* < 0.05), whereas no significant change was detected in the corresponding short-term group (*p* > 0.05).

As shown in Figure 1b, *hsp70* expression was significantly downregulated in the short-term 0.5% and 1% FeSO_4_ groups (*p* < 0.05). In contrast, long-term exposure to the same concentrations resulted in significant upregulation of *hsp70* expression (*p* < 0.05).

As shown in Figure 1c, *ferritin* expression was significantly upregulated in the short-term 0.5% and long-term 1% FeSO_4_ groups (*p* < 0.05). No significant differences were observed in the other treatment groups compared to the control (*p* > 0.05).

As shown in Figure 1d, *sod* expression was significantly upregulated in the short-term 1% and long-term 0.5% and 1% FeSO_4_ groups (*p* < 0.05). In contrast, a significant downregulation was observed in the short-term 0.5% FeSO_4_ group (*p* < 0.05).

As shown in Figure 1e, *c3* expression was significantly downregulated in the short-term 0.5% FeSO_4_ group (*p* < 0.05). In contrast, significant upregulation was detected in the short-term 1% and long-term 0.5% groups (*p* < 0.05), while no significant change was observed in the long-term 1% FeSO_4_ group (*p* > 0.05).

As shown in Figure 1f, *tlr3* expression was significantly downregulated across all FeSO_4_ treatment groups (*p* < 0.05). Among them, the short-term 0.5% FeSO_4_ group exhibited relatively higher expression levels than the other treatments (*p* < 0.05), despite the overall suppressive effect.

## 4. Discussion

### 4.1. Long-Term Iron Supplementation Enhances Sea Cucumber Resistance to Pathogen Infection

This study examined the immunological and antioxidant responses of *A. japonicus* to dietary ferrous sulfate (FeSO_4_) supplementation under *V. splendidus* infection, with a focus on gene expression and enzyme activity changes. The results showed that long-term supplementation with 1% FeSO_4_ significantly improved the survival rate of infected sea cucumbers, while no significant changes were observed in other groups. This indicates that continuous dietary FeSO_4_ supplementation, particularly at a concentration of 1%, can notably enhance the resistance of *A. japonicus* to *V. splendidus* infection. In contrast, short-term supplementation improved certain immune parameters but did not significantly increase survival, suggesting that short-term stimulation may initiate a preliminary immune response but lacks sufficient duration to achieve effective protection. Notably, the control groups exhibited high variability in survival rates (large SD values), which likely reflects individual differences in baseline stress tolerance and susceptibility to infection. In contrast, survival in the long-term 1% FeSO_4_ group was higher and more consistent, as reflected by the lower SD. This suggests that FeSO_4_ supplementation had a stabilizing effect in addition to improving survival.

Phagocytes are a key component of the sea cucumber’s immune system, responsible for recognizing and engulfing pathogens and directly contributing to pathogen clearance [31]. FeSO_4_ supplementation may influence phagocytic activity and respiratory burst by providing bioavailable iron, crucial for activating iron-dependent enzymes like NADPH oxidase. This promotes ROS production and enhances the immune cells’ ability to kill pathogens [32,33]. In this study, long-term FeSO_4_ supplementation significantly increased phagocytic activity, with the 1% FeSO_4_ group showing the most pronounced enhancement, whereas short-term supplementation at both concentrations had no significant effect. This suggests that long-term FeSO_4_ intake improves the phagocyte-mediated clearance of pathogens. Similar findings have been reported in echinoderms, where coelomocyte phagocytosis was strongly linked to pathogen resistance in sea cucumbers and sea urchins [10,34].

Respiratory burst activity, mainly mediated by coelomocytes, is a key component of the sea cucumber immune system. It produces reactive oxygen species (ROS), such as superoxide anion and hydrogen peroxide, to kill invading microbes [35,36]. In this study, respiratory burst activity was markedly enhanced in the long-term 1% FeSO_4_ group, whereas both short-term supplementation groups exhibited unexpectedly low levels. This paradoxical decline may reflect the time-dependent nature of immune modulation. Short-term supplementation may not allow sufficient incorporation of iron into coelomocyte metabolism to effectively activate ROS-generating pathways. Moreover, sudden increases in intracellular iron can trigger compensatory antioxidant defenses to prevent oxidative damage, resulting in transient suppression of respiratory burst activity [37,38]. In addition, iron may initially be preferentially allocated to storage or repair functions rather than immune effector pathways [39]. These findings suggest that the reduced respiratory burst after short-term FeSO_4_ supplementation is a transient adaptive adjustment. In contrast, long-term supplementation leads to a stable enhancement of ROS-mediated immune defense. Similar studies in sea urchins have found that higher respiratory burst capacity is associated with stronger pathogen resistance. Disease-resistant sea urchins produce more reactive oxygen species (ROS) than their susceptible counterparts [34].

Phagocytosis and respiratory burst often work synergistically in immune defense. Together, they form an efficient cellular immune system that plays a crucial role in combating pathogenic infections [40,41]. Therefore, long-term FeSO_4_ supplementation that strengthens both mechanisms may effectively promote the clearance of *V. splendidus*. It is important to note, however, that excessive ROS generated during respiratory burst can cause oxidative damage to host cells. Thus, the antioxidant system must regulate ROS levels to prevent oxidative stress and maintain cellular integrity [42,43].

### 4.2. Synergistic Regulation of Oxidative Stress and the Antioxidant Defense System Enhances Host Immune Resilience

In terms of antioxidant defense, SOD activity was significantly elevated in the long-term 1% FeSO_4_ group, with a moderate increase observed in the short-term 1% group. No significant changes were found in either the short- or long-term 0.5% FeSO_4_ groups. Iron is a vital component of antioxidant enzymes like SOD, and FeSO_4_ supplementation provides bioavailable Fe^2+^, essential for activating SOD. This increased iron availability enhances SOD’s ability to catalyze the conversion of superoxide anions into hydrogen peroxide and oxygen, thereby reducing oxidative damage to cells [44,45]. Importantly, *sod* gene expression was also significantly upregulated in these groups, consistent with the enzyme activity results. This cross-reference between transcriptional regulation and enzymatic function indicates that FeSO_4_ supplementation enhanced antioxidant defense at both molecular and functional levels. Furthermore, respiratory burst activity was enhanced alongside increased SOD activity, which indicates that FeSO_4_ supplementation may support both ROS production for microbial killing and antioxidant defenses to reduce potential oxidative damage. This balance between ROS production and SOD activity ensures that ROS remain at levels sufficient to kill pathogens while preventing excessive oxidative damage to host cells [46]. While this pattern is consistent with a functional balance between pro-oxidant and antioxidant responses, the evidence here is indirect, and further studies would be required to confirm the underlying mechanisms [47].

The *hsp70* gene encodes the molecular chaperone heat shock protein 70 (Hsp70), which helps maintain protein homeostasis under stress and protects cells from damage [48]. In this study, *hsp70* expression was significantly increased in the long-term FeSO_4_ groups, indicating enhanced cellular stress tolerance, which may help immune cells maintain functional stability under oxidative pressure [49,50].

The *ferritin* gene encodes the main iron-storage protein in *A. japonicus*, which regulates iron metabolism and storage to maintain iron homeostasis [18]. In our study, *ferritin* expression was significantly upregulated in the short-term 0.5% group and the long-term 1% group, suggesting that FeSO_4_ supplementation activated iron storage mechanisms. This may reduce the risk of Fenton reactions by sequestering excess Fe^2+^ ions [51]. Ferritin, in cooperation with SOD and other antioxidant enzymes, contributes to intracellular iron regulation and free radical scavenging, thus alleviating potential oxidative damage caused by iron supplementation [52].

Collectively, these results demonstrate that FeSO_4_ supplementation enhances both oxidative bactericidal activity and antioxidant capacity in *A. japonicus*. Long-term supplementation, particularly at 1%, yielded the most pronounced effects, with improved phagocytosis, respiratory burst, and higher expression of *sod*, *ferritin*, and *hsp70*. This suggests that higher iron levels may exceed the activation threshold for cellular and oxidative defenses, shifting the immune response toward these mechanisms. This aligns with studies showing that iron modulates innate immune pathways in a dose-dependent manner and that elevated iron can trigger ferroptosis-related defenses in sea cucumbers [17,39]. Moreover, research on immunonutrition indicates that while moderate supplementation boosts immune resilience, excessive doses may skew immune responses towards cellular and oxidative pathways [53].

### 4.3. Humoral Immune Regulation and Associated Gene Expression Mechanisms

LZM degrades bacterial cell walls and works synergistically with phagocytosis to enhance antimicrobial effects [54]. The short-term supplementation with 0.5% and 1% FeSO_4_ significantly increased LZM activity, indicating an enhanced humoral immune response. FeSO_4_ supplementation provides bioavailable iron (Fe^2+^), which is essential for the optimal activation of immune enzymes such as LZM. Iron may enhance the synthesis and secretion of LZM by immune cells, potentially by regulating gene expression and activating associated signaling pathways [39,55]. In the long-term trial, the 0.5% FeSO_4_ group maintained high LZM levels, while no significant change was observed in the 1% FeSO_4_ group. This discrepancy suggests a non-linear dose–response pattern for LZM activity. Moderate supplementation (0.5%) may optimally stimulate humoral immunity, while higher concentrations may trigger regulatory mechanisms or feedback inhibition. Similar non-linear responses to immunostimulants have been reported in aquaculture studies [53].

Interestingly, under short-term feeding with 0.5% FeSO_4_, the expression of the *c3* gene significantly decreased, while no significant changes were observed in the long-term 1% FeSO_4_ group. This suggests that the complement system is sensitive to both Fe^2+^ concentration and exposure duration, with differential expression possibly caused by oxidative stress, immune feedback inhibition, or indirect effects of iron metabolism on transcriptional activity [56].

C3 cleavage products (e.g., C3b) can label pathogens, promoting their recognition and phagocytosis by phagocytes, thereby enhancing immune clearance efficiency [57,58]. Meanwhile, LZM directly kills bacteria by disrupting their cell walls, both mechanisms working together with phagocytosis to form a robust immune defense [59]. Previous studies have shown that upregulation of *c3* and *lyz* expression correlates with increased phagocytic capacity and overall immune activation [60,61]. Taken together, these results suggest a dose-dependent divergence in immune modulation. Long-term supplementation with 0.5% FeSO_4_ promoted higher LZM activity and *c3* expression, suggesting a preference for soluble effector molecules that provide rapid extracellular defense while minimizing oxidative stress. In contrast, 1% FeSO_4_ appeared to enhance cellular and antioxidant responses, including phagocytosis and ROS-related pathways. The findings suggest that the two dosages operate through distinct but complementary immune pathways.

At the gene expression level, the *lyz* gene and LZM enzyme activity showed partially inconsistent patterns, particularly in the short-term 1% group where transcription was downregulated but enzyme activity increased. This discrepancy suggests that lysozyme regulation in *A. japonicus* may not be determined solely at the transcriptional level, but is also influenced by post-transcriptional regulation, protein stability, or secretion processes. In contrast, in the long-term 0.5% FeSO_4_ group, both *lyz* expression and LZM activity were elevated, indicating that moderate supplementation can stimulate humoral immunity at both transcriptional and enzymatic levels. However, the divergence observed in the 1% groups highlights that enzyme activity can be maintained or even enhanced despite transcriptional downregulation. Similar mismatches between *lyz* gene expression and enzymatic activity have been reported in sea cucumbers under salinity stress and in mollusks under immune challenges [60,62]. This supports the notion that such discrepancies are common among invertebrates. These findings highlight the complexity of humoral immune regulation in *A. japonicus*. They suggest that FeSO_4_ dosage influences LZM activity through multiple regulatory layers, from gene transcription to post-transcriptional and protein-level mechanisms.

AKP and ACP are key enzymes in the immune system of *A. japonicus*, involved in the degradation of pathogenic cell walls and the transmission of immune signals [63,64]. These enzymes enhance lysosomal function in phagocytes and improve the efficiency of pathogen clearance. In this study, short-term FeSO_4_ supplementation significantly increased ACP and AKP activities. This suggests that FeSO_4_ can rapidly activate the non-specific immune response in *A. japonicus*, enhancing its initial defense against exogenous pathogens [65]. In contrast, enzyme activities decreased with long-term supplementation, possibly reflecting a negative regulatory mechanism by the host to prevent immune overactivation after prolonged stimulation. Similar changes occur in other aquatic animals, like crucian carp and common carp, where ACP and AKP expression are influenced by the intensity and duration of immune stress. This may represent an adaptive immune homeostasis mechanism in response to FeSO_4_ exposure [66,67].

Toll-like receptor 3 (TLR3) is a pattern recognition receptor. It identifies specific molecular patterns from pathogens and activates downstream signaling pathways to initiate immune responses [68]. In the present study, expression of the *tlr3* gene was significantly downregulated in all FeSO_4_-treated groups. Previous studies have shown that excessive expression of *tlr3* in aquatic animals, including echinoderms, may lead to chronic inflammation or tissue damage [57,69]. Thus, moderate downregulation of *tlr3* may act as a protective mechanism to prevent immune overactivation and maintain homeostasis. The FeSO_4_-induced *tlr3* suppression observed in this study may serve as a negative feedback strategy to balance antimicrobial activity and immune stability.

## 5. Conclusions

In conclusion, short-term FeSO_4_ supplementation enhances humoral immunity and increases the activity of lysosomal enzymes, including LZM, ACP, and AKP. By contrast, long-term FeSO_4_ supplementation strengthened immune defense and oxidative stress management in sea cucumbers, increasing phagocytic activity, respiratory burst, SOD activity, and the expression of stress-related proteins like *ferritin* and *hsp70*. The synergistic enhancement of cellular immunity, humoral factors (e.g., LZM, C3), and antioxidant enzymes enables sea cucumbers to generate sufficient antimicrobial ROS while simultaneously limiting oxidative damage, thereby improving resistance against *V. splendidus.*

From a practical perspective, these findings suggest that dietary supplementation with FeSO_4_ at around 1% for at least 56 days represents an effective strategy to enhance the health and disease resistance of *A. japonicus* in aquaculture. Moderate supplementation (0.5%) may also provide benefits by stimulating humoral immunity, albeit with weaker protection. However, potential FeSO_4_ toxicity at higher doses and the long-term safety of supplementation remain important considerations for future studies. Additionally, the applicability of FeSO_4_ supplementation in field farming conditions should be further evaluated for cost-effectiveness and practical implementation. Multi-omics approaches, such as RNA-seq, should be used to clarify the molecular pathways of dose-dependent immune modulation and explore potential interactions with the host microbiome.

## Figures and Tables

**Table 1:** Primer sequences for qPCR.

Gene	Primer Sequence (5′→3′)	NCBI Accession No.	Product Size (bp)
*β-actin* [26]	F:TTATGCTCTTCCTCACGCTATCC R:TTGTGGTAAAGGTGTAGCCTCTCTC	EU668024	132
*tlr3* [27]	F:TTGAAGCGTTGGATTTG R:GGACCGATGTTGGAGATA	XM071957083	124
*hsp70* [28]	F:ATGCCTAGAACCAGTAGAGAAAG R:TGTCGTTCGTGATGGTGATT	GH985449	116
*Lyz* [29]	F:GTGTCTGATGTGGCTGTGCT R:TTCCCCAGGTATCCCATGAT	EF036468	146
*c3*	F: GGGAAGGCATTCACAAGACA R: CAACCCAGCTTGCTTGAAC	HQ214156	232
*sod*	F:CACTTCAGCAGGGGGACATT R:GAGGGCCGGATAGCGATATG	JF769856	158
*ferritin*	F: TCCTGGAGCCCACAAGTACT R: TTTCTGGAGCCGTGATGTCC	DQ058411	125

**Table 2:** Survival Rate, Phagocytic Cell Activity, and Respiratory Burst Activity.

Group	ST Control	LT Control	ST 0.5% FeSO_4_	ST 1% FeSO_4_	LT 0.5% FeSO_4_	LT 1% FeSO_4_
Survival Rate (%)	50 ± 10 ^a^	53 ± 12.5 ^a^	66.7 ± 4.7 ^a^	73.3 ± 12.5 ^a^	80 ± 8.2 ^a^	90 ± 4.7 ^b^
Phagocytic Activity (OD540/10^6^ cells)	0.45 ± 0.23 ^a^	0.54 ± 0.15 ^a^	0.41 ± 0.09 ^a^	0.40± 0.17 ^a^	0.74 ± 0.03 ^ab^	1.02 ± 0.06 ^b^
Respiratory Burst (OD630/10^6^ cells)	0.42 ± 0.07 ^ab^	0.49 ± 0.16 ^ab^	0.11 ± 0.03 ^c^	0.18 ± 0.07 ^bc^	0.49 ± 0.13 ^a^	0.87 ± 0.08 ^e^

Note: ST = Short-term feeding; LT = Long-term feeding; Data in the same row with different lowercase letters indicate significant differences (*p* < 0.05). The same applies for below.

**Table 3:** Immune and Antioxidant-Related Enzyme Activities.

Group	ST Control	LT Control	ST 0.5% FeSO_4_	ST 1% FeSO_4_	LT 0.5% FeSO_4_	LT 1% FeSO_4_
AKP (G/100 mL)	1.35 ± 0.09 ^a^	1.30 ± 0.09 ^a^	2.87 ± 0.39 ^b^	2.47± 0.36 ^c^	0.60 ± 0.04 ^d^	0.60 ± 0.11 ^d^
ACP (G/100 mL)	1.39 ± 0.31 ^a^	1.41 ± 0.15 ^a^	2.60 ± 0.18 ^b^	2.31 ± 0.30 ^b^	0.50 ± 0.30 ^c^	0.55 ± 0.55 ^c^
SOD (U/mL)	82.97 ± 2.12 ^a^	81.58 ± 1.77 ^a^	85.27 ± 3.96 ^a^	91.44 ± 5.65 ^b^	84.04 ± 2.00 ^a^	95.22 ± 2.30 ^c^
LZM (μg/mL)	1.38 ± 0.16 ^a^	1.43 ± 0.6 ^a^	1.68 ± 0.11 ^b^	1.69 ± 0.05 ^b^	1.88 ± 0.05 ^c^	1.44 ± 0.19 ^a^

Note: ST = Short-term feeding; LT = Long-term feeding; Data in the same row with different lowercase letters indicate significant differences (*p* < 0.05). The same applies for below.

## Data Availability

All of the data generated or analyzed during this study are included in this published article.
